# Supplementary material for: ModDotPlot—Rapid and interactive visualization of complex repeats
Source: bioRxiv. 2024 Apr 19:2024.04.15.589623. Preprint. [Version 1] doi: 10.1101/2024.04.15.589623 (PMC11071298; doi:10.1101/2024.04.15.589623)
Supplement: Supplement 1 [file media-1.pdf]

Supplemental Material

Algorithm 1 Partition Sequence S into sets of modimizers

Input: List of hashed k-mers $S_k[x_1, x_2, ...x_n]$, window size w, sparsity s
Output: List of modimizer sets $MOD_s = [A_1, A_2, ..., A_r]$

function BASELAYER(S_k,w,s)
    $n \leftarrow$ length of S_k
    $r \leftarrow \lceil \frac{n}{w} \rceil$ ▷ Set number of windows (resolution) based on n and w
    $m \leftarrow \frac{w}{s}$ ▷ Set the expected modimizer density
    $MOD_s \leftarrow$ list of size r
    for $i \leftarrow 0$ to r **do** ▷ Retrieve all modimizers within each interval
        $MOD_s[i] \leftarrow \{\}$
        $start \leftarrow wi$ ▷ Set non-overlapping interval boundaries
        $end \leftarrow \min((start + w), n - 1)$
        $MOD_s[i] \leftarrow$ GETMODIMIZERS$(S_k[start : end], s, d)$ ▷ Populate list with sets of modimizers
    end for
    return MOD_s
end function

function GETMODIMIZERS(S_k, s, m)
    $A \leftarrow \forall x \in \{S_k[A]\} : x \equiv 0 \text{ mod } s$ ▷ Gather the set of unique modimizers per interval
    $\hat{s} \leftarrow \frac{s}{2}$
    while $|A| < \frac{m}{2}$ and $\hat{s} > 1$ **do** ▷ Resample at higher density if number of modimizers is under threshold
        $A \leftarrow \forall x \in \{S_k[A]\} : x \equiv 0 \text{ mod } \hat{s}$
        $\hat{s} \leftarrow \frac{\hat{s}}{2}$
    end while
    return A
end function

Algorithm 2 Partition Sequence S into a modimizer hierarchy H

Input: List of hashed k-mers $S_k[x_1, x_2, ...x_n]$, minimum window size $\hat{w}$, sparsity $\hat{s}$, resolution r
Output: List $H = [MOD_{\hat{s}}, MOD_{2\hat{s}}, ..., MOD_{2^{l-1}\hat{s}}]$

function BUILDHIERARCHY(S_k,$\hat{w}$,$\hat{s}$,r)
    $n \leftarrow$ length of S_k
    $l \leftarrow \lfloor \log_2(\frac{n}{\hat{w}r}) \rfloor$ ▷ Initialize number of layers based on min. window size and resolution
    $H \leftarrow$ list of size l
    $H[0] \leftarrow$ BASELAYER$(S_k, \hat{w}, \hat{s})$ ▷ Compute bottom layer
    for $i \leftarrow 1$ to l **do** ▷ Iteratively compute subsequent layers from previous layer
        $\hat{r} \leftarrow 2^{l-1-i}r$ ▷ Halve the resolution when building subsequent layer
        $H[i] \leftarrow$ ADDLAYER$(H[i-1], 2^i\hat{s}, 2^i\hat{w}, n, \hat{r})$
    end for
    return H
end function

function ADDLAYER($MOD_{\hat{s}}[A_1, A_2, ..., A_r], s, w, n, \hat{r}$)
    $MOD_s \leftarrow$ list of size $\hat{r}$ ▷ Initialize current layer
    $m \leftarrow \frac{w}{s}$ ▷ Update expected sketch size for current layer
    for $i \leftarrow 0$ to $\hat{r}$ **do** ▷ Retrieve modimizers from matching intervals within the previous layer
        $MOD_s[i] =$ GETMODIMIZERS$((MOD_{\hat{s}}[A_{2i}] \cup MOD_{\hat{s}}[A_{2i+1}]), s, m)$
    end for
    return MOD_s
end function

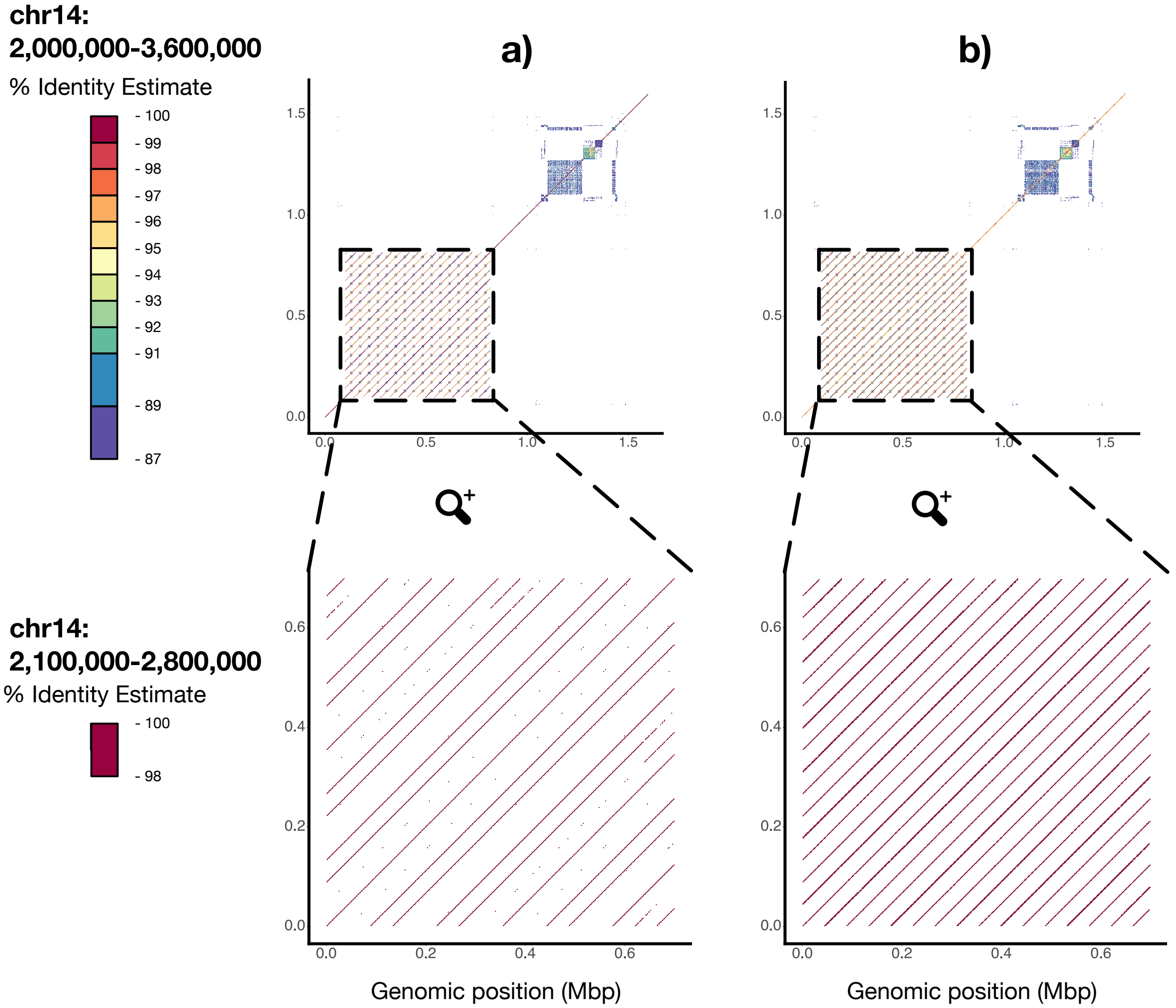

Supplementary Figure 1. Screenshots of ModDotPlot run on a human acrocentric short arm (CHM13 chr14:2,000,000-3,600,000), highlighting the rDNA array with and without ModDotPlot's interval extension activated. **a)** With no interval extension, the full 16 copy rDNA array is visible; however, when zoomed in (chr14:2,100,000-2,800,000) and filtered for >98% sequence identity, some rDNA copies disappear from the plot due to registration artifacts. **b)** With intervals extended (i.e. when computing similarity for the cell M(A,B), interval B is extended by $w/2$ in both directions), all rDNA copies appear at all zoom levels.

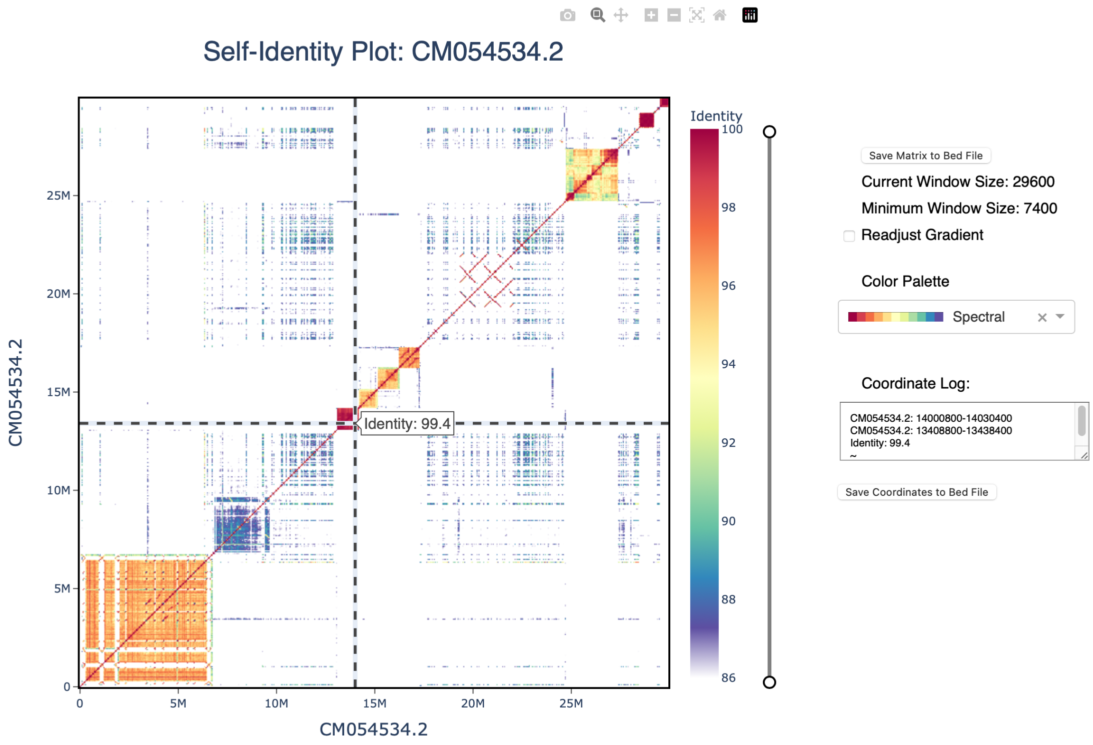

Supplementary Figure 2. Screenshot of ModDotPlot's interactive mode, showcasing the entire Y chromosome of a gibbon (mSymSyn1 (1)). Despite spanning almost 30 Mbp, ModDotPlot was able to create 3 matrices in under 2 minutes, with around 2.5GB of memory. Screenshot was taken using ModDotPlot version v0.8.0 (git commit `ed190c7`).

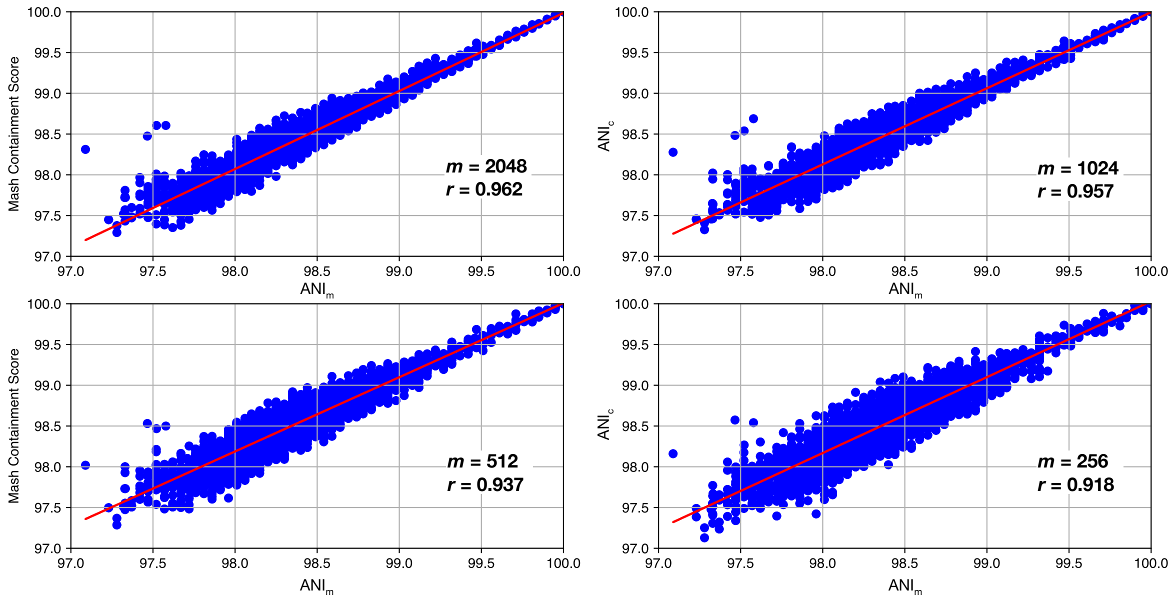

Supplementary Figure 3. Scatterplot of ANI_m against the ANI_c distances under various sketch sizes. The 10-mer non-canonical HOR region from Figure 4 was excluded from analysis.

References

1. Kateryna D. Makova, Brandon D. Pickett, Robert S. Harris, Gabrielle A. Hartley, Monika Cechova, Karol Pal, Sergey Nurk, DongAhn Yoo, Qiuhui Li, Prajna Hebbar, Barbara C. McGrath, Francesca Antonacci, Margaux Aubel, Arjun Biddanda, Matthew Borchers, Erich Bornberg-Bauer, Gerard G. Bouffard, Shelise Y. Brooks, Lucia Carbone, Laura Carrel, Andrew Carroll, Pi-Chuan Chang, Chen-Shan Chin, Daniel E. Cook, Sarah J.C. Craig, Luciana de Gennaro, Mark Diekhans, Amalia Dutra, Gage H. Garcia, Patrick G.S. Grady, Richard E. Green, Diana Haddad, Pille Hallast, William T. Harvey, Glenn Hickey, David A. Hillis, Savannah J. Hoyt, Hyeonsoo Jeong, Kaivan Kamali, Sergei L. Kosakovsky Pond, Troy M. LaPolice, Charles Lee, Alexandra P. Lewis, Yong-Hwee E. Loh, Patrick Masterson, Kelly M. McGarvey, Rajiv C. McCoy, Paul Medvedev, Karen H. Miga, Katherine M. Munson, Evgenia Pak, Benedict Paten, Brendan J. Pinto, Tamara Potapova, Arang Rhie, Joana L. Rocha, Fedor Ryabov, Oliver A. Ryder, Samuel Sacco, Kishwar Shafin, Valery A. Shepelev, Viviane Slon, Steven J. Solar, Jessica M. Storer, Peter H. Sudmant, Sweetalana, Alex Sweeten, Michael G. Tassia, Françoise Thibaud-Nissen, Mario Ventura, Melissa A. Wilson, Alice C. Young, Huiqing Zeng, Xinru Zhang, Zachary A. Szpiech, Christian D. Huber, Jennifer L. Gerton, Soojin V. Yi, Michael C. Schatz, Ivan A. Alexandrov, Sergey Koren, Rachel J. O'Neill, Evan E. Eichler, and Adam M. Phillippy. The complete sequence and comparative analysis of ape sex chromosomes. *bioRxiv*, 2024.
